# Who is researching biodiversity hotspots in Eastern Europe? A case study on the grasslands in Romania

**DOI:** 10.1371/journal.pone.0217638

**Published:** 2019-05-28

**Authors:** Andreea Nita, Tibor Hartel, Steluta Manolache, Cristiana M. Ciocanea, Iulia V. Miu, Laurentiu Rozylowicz

**Affiliations:** 1 Center for Environmental Research and Impact Studies, University of Bucharest, Bucharest, Romania; 2 Department of Biology and Ecology in Hungarian and Center for Systems Biology, Biodiversity, and Bioresources, Babes-Bolyai University, Cluj-Napoca, Romania; 3 Chelonia Romania, Bucharest, Romania; Helmholtz Centre for Environmental Research - UFZ, GERMANY

## Abstract

European farmlands are vital arenas for socio-ecological sustainability because of their significant land coverage and potential for integrating food production with biodiversity conservation. The knowledge produced by scientific research is a critical ingredient in developing and implementing socio-economically and ecologically sustainable management strategies for farming landscapes. The grasslands of Europe have been managed for millennia. They have exceptional socio-cultural and economic value and are among the most biodiverse ecosystems in the world. The quality of scientific knowledge on them and its potential to address grasslands as complex socio-ecological systems is strongly dependent not only on the creativity and scientific ambition of the researcher, but also on the network around the researcher (including both academic and non-academic sectors). The goal of this study is to map the research network around Romania’s grasslands using bibliometrics analysis, a well-developed scientific domain that utilizes network theory to analyze relationships between affiliations networks, co-authorship networks, and co-word analysis. The number of studies targeting grasslands in Romania is increasing, owing mostly to international involvement. However, management of the grasslands is still deficient and the contribution of science to the process is virtually absent. The current research is mainly related to the biological and ecological characteristics of grasslands, with topics related to their management notably absent from internationally visible research, especially in the context of EU Common Agricultural Policies. To increase scientific inquiry and better inform the EU and local policies on grasslands management, Romanian researchers should capitalize on international collaborations and local academic leaders. Our findings can be used to identify research gaps and to improve collaboration and knowledge exchange between practitioners, researchers, policy makers, and stakeholders.

## Introduction

The integration of agricultural production, biodiversity conservation, and socio-cultural values is a key challenge in promoting the sustainability of socio-ecological systems [1]. The current grasslands of Europe were developed through millennia of management by humans, which typically included allowing livestock to graze and producing hay. Grasslands have made a substantial contribution to the high nature value (HNV) farmlands of the European Union [2–4]. Several protected species and habitats are linked to grasslands and are dependent on some form of extensive, multifunctional management of these ecosystems [5, 6]. Furthermore, wooded meadows and wood-pastures are considered an archetypical manifestation of HNV farmlands in Europe [7].

Many EU countries have already lost culturally and naturally important grasslands due to changes in management practices [8]. The intensive, highly specialized management of grasslands has resulted in a sharp decrease in their biodiversity, aesthetic, and cultural value, and even their disappearance in some places. Land abandonment brings opportunities and threats, both in terms of wildlife species and ecosystem services provision [9–12].

Knowledge is essential for promoting socio-economically and ecologically sustainable grasslands management [1]. Scientific research can generate a contextual understanding of the relationship between the management intensity and the biodiversity and productivity of the grasslands [13]. On the other hand, scientific research is important in developing new types of conceptualizations of the grasslands, for example, as complex, adaptive systems that can exist in multiple socio-ecological states [14, 15]. Nevertheless, knowledge can contribute to re-addressing current management paradigms around the production landscapes [16], including grasslands. Since grasslands can simultaneously serve multiple socio-ecological functions and roles (e.g., production, biodiversity conservation, and recreational and cultural values), an overly narrow (e.g., disciplinary) scientific approach for understanding them tends to promote simplistic management measures that often lack socio-cultural contextualization. The quality of scientific knowledge and its potential to address grasslands as complex socio-ecological systems are strongly dependent not only on the creativity and scientific ambition of the researcher, but also on the social network (including both academic and non-academic sectors) of the researcher [17].

In this study, we address the collaboration network between academics and the diversity of research fields related to Romanian grasslands. The importance of this research is threefold. First, Romanian grasslands are among the hotspots of vascular plant species richness [18], encompassing several ancient land-uses and practices, such as wood-pastures [7, 19], traditional stewardship, and management [20]. These grasslands are currently under threat from overgrazing, changes in management and stewardship, and abandonment [15, 21–23]. Second, Romania is a developing country in Eastern Europe, where research is suffering from lack of funds, institutional instability, and intense political pressure [24, 25]. This overall harsh conjuncture for research is hampering the production of holistic knowledge [26] and innovation, which will be indispensable to Romania as it navigates the challenges of globalization, including its increasing role in ensuring food security [27] and the need to cope with extreme climate variations [28]. Third, the academic world in Romania is still transitioning from local/regional, disciplinary thinking to the adoption of international standards of scientific rigor and holistic, inter-, and trans-disciplinary approaches. Because of this, Romania is sharply underrepresented in the international scientific databases, while publishing in local journals (i.e., those hosted by Romanian academic institutions) is still actively promoted in academia. Overall, the management of grasslands in Romania is still deficient [29] despite the latest legal motions, which most often do not consider the contribution of science to the process.

Collaboration between academics and knowledge creation have been extensively studied using network analysis framework. Analyses span from understanding patterns of scientific collaboration resulting from co-authorship and institutional networks [30–32] to knowledge creation [33], prediction of productivity [34] or trends [35]. In this paper, we use network analysis to characterize the status of scientific research in the field of grasslands governance in Romania and to suggest ways to improve scientific understanding by 1) revealing internationally visible research on Romania’s grasslands published after 1990; 2) highlighting the most important institutions generating research and mapping the invisible authors and academic leaders of the co-authorship network; and 3) analyzing the keywords co-occurrence network to discover the most common keywords and topics of scientific interest.

## Methods

**Fig 1:**
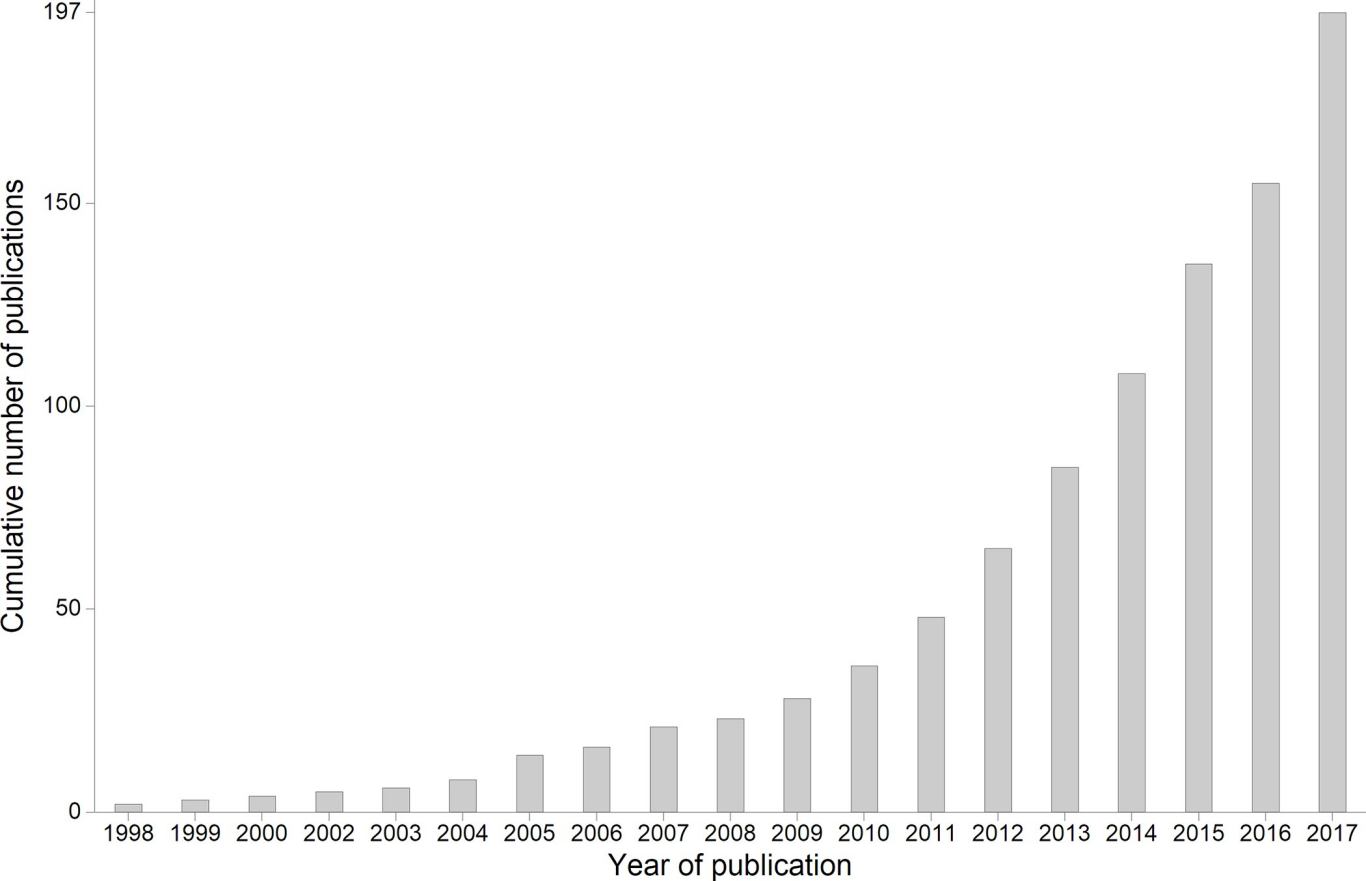
Cumulative number of publications targeting Romania’s grasslands that are accessible in the Scopus database (1998–2017).

To identify the research network around Romania’s grasslands, we extracted the literature that simultaneously had one of the following keywords in the abstract, title, or the list of keywords from the Scopus database (Elsevier B.V.) and adding *Romania* to each of them (e.g., “pasture” AND “Romania”): *Common Agricultural Policy*, *CAP*, *pasture*, *grassland*, *meadow*, *lawn*, *greensward*, *grazing*, *graze*, *silvopastoral*, *pastureland*, *rangeland*, and *mowing*. We obtained 602 articles, book chapters, and documents recording conference proceedings that might potentially be related to the subject under investigation. We reviewed each of the identified publications and removed those published before 1990 as well as those that did not contain information about the subjects of our review (e.g., paleoecology, paleobotany). The final database included 197 publications investigating topics related to Romanian grasslands (i.e., the effects of different types of management, ecological traits and community structure, conservation and improvement measures for these habitats, etc.), from which we extracted 173 peer-reviewed articles and 24 recordings of proceedings (hereinafter known as “articles”) (Fig 1). For each article, we extracted the list of keywords, authors, and their first affiliation (as stated in the papers). Fig 1 shows an ascending trend in the number of publications, which can be interpreted as an indicator of the increasing interest in Romanian grasslands among scientific researchers.

The peer-reviewed articles targeting Romania’s grasslands were published in 107 journals and recordings of proceedings. The top journals in our network, with more than 5 publications, were *Quality-Access to Success* (15 publications), *International Multidisciplinary Scientific GeoConference Surveying Geology and Mining Ecology Management* (SGEM) (14), *Notulae Botanicae Horti Agrobotanici Cluj-Napoca* (8), *PLoS ONE* (6), *Applied Vegetation Science* (5), *Biodiversity and Conservation* (5), the *Environmental Engineering and Management Journal* (5), and the *North-Western Journal of Zoology* (5).

**Fig 2:**
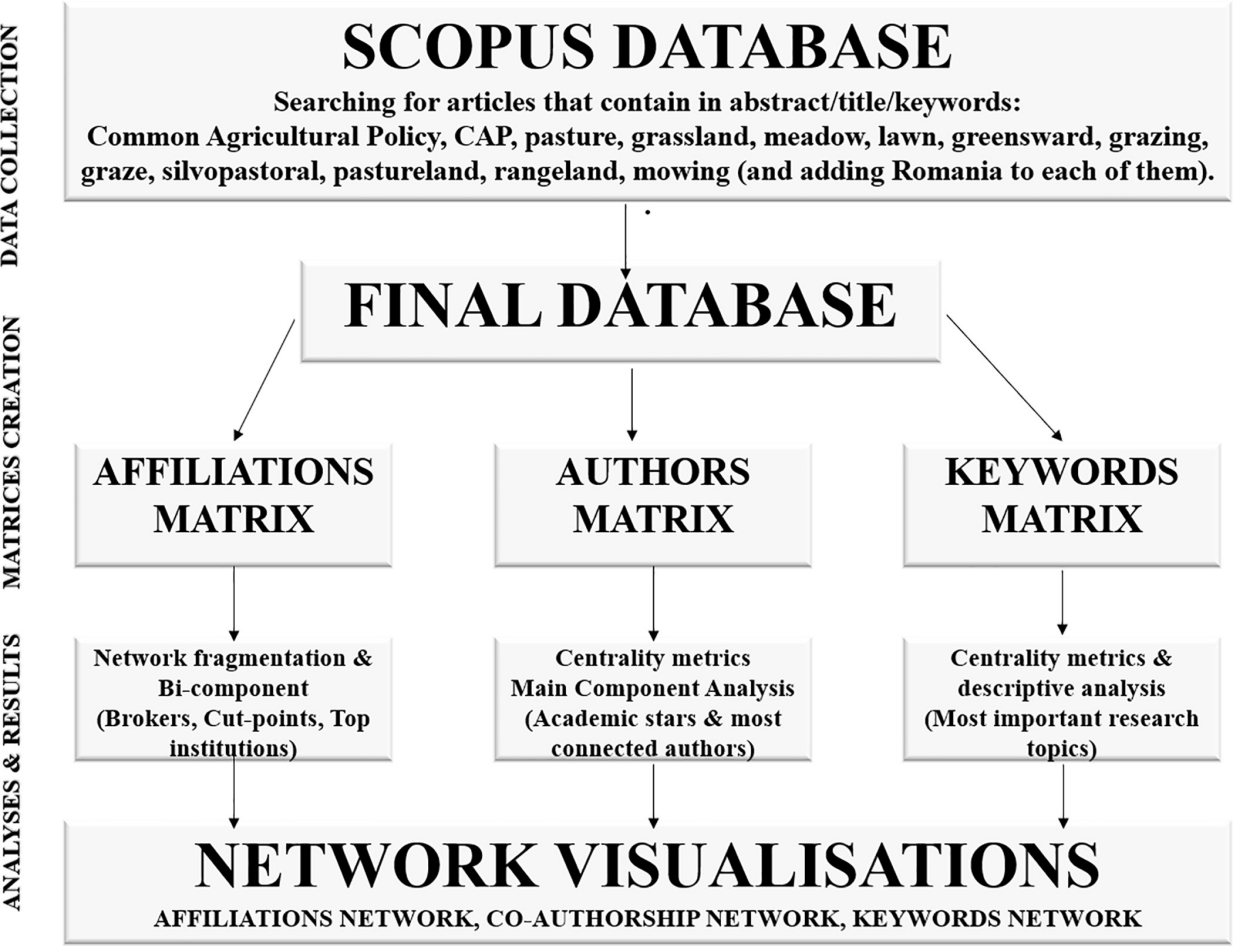
Workflow of network analyses of internationally visible research related to grasslands in Romania.

For the bibliometric analysis, we constructed three network matrices: (i) an affiliations network, to make inferences about inter-institutional cooperation; (ii) a co-authorship network, which highlights invisible authors and academic leaders; and (iii) and a co-occurrence keywords network to discover hidden connections between the most common keywords, research topics, and topics of scientific interest. For these analyses, we created distinct databases which were cleaned and unified (i.e., standardizing the names of institutions, authors, and keywords to avoid duplication of entries due to different spellings). The cleaned matrices included 192 unique affiliations (516 entries initially), 517 unique authors (755 entries initially), and 577 unique keywords (1019 entries initially). The methodology workflow is presented in Fig 2.

### Affiliations analysis

We used *bi-component analysis* to identify blocks (bi-connected subnetworks) and the cut-points (articulation points) in the affiliation graph [36]. If removed, cut-point institutions break the affiliation network into one or more bi-connected subnetworks [37]. Such institutions are important for the cohesion of the research network focused on Romanian grasslands and might act as research brokers among otherwise disconnected groups [38]. An affiliation subgraph is bi-connected if every institution in the subgraph (at least three) has direct connections to the others and the subgraph would remain connected even if a node were removed [36]. The number and size of blocks is an indication of network fragmentation. If a network is dominated by one block, then most institutions cooperate during the research. Smaller blocks include isolated institutions (i.e., research institutions that publish without collaborating with other organizations) that published a small number of papers on the subject or papers on uncommon topics [37].

We also calculated several node-level centrality metrics: degree, betweenness, and eigenvector. The *degree centrality* of an institution represents the number of direct connections [38, 39] and helps to identify the most collaborative institutions (i.e., the organizations with the highest number of connections to other research units in Romanian grasslands research). The metric does not account for the importance of the institutions that are linked to the node of interest. Thus, an institution can be considered as collaborative even if it is linked only to organizations that have no other collaborations in the network. The *eigenvector centrality* is like degree centrality but assigns higher scores to connections with institutions that are themselves well-connected [38]. This metric represents the sum of the eigenvectors of the institutions that the institution of interest is connected to, and it reveals the best options for future partnerships (the most influential institutions, which can further promote partnership in research) [38]. The *betweenness centrality* measures the extent to which a research body lies on paths between other institutions from the research network. Such organizations can control the flow of information in the network if we assume that every pair of connected institutions exchanges information with equal probability and that information flow on a short path is chosen at random [40]. They may be considered bridge institutions (i.e., they can control the research subjects in a partnership) [37]. Relationships among countries and within countries, as demonstrated by the affiliations declared by the authors, were represented using a chord diagram [41].

### Analysis of authors

We use an authors’ matrix to analyze the co-authorship network surrounding Romanian grasslands research and to illustrate the patterns of cooperation between the scientists in this field of research [34, 42]. We calculated the *network fragmentation* metric, which (in our case) indicates the proportion of pairs of authors who cannot reach each other [38]. Large datasets, as in the case of our database, typically involve many small independent clusters around larger ones and one large and dense cluster [31]. Thus, we assessed the distribution of components in the co-authorship network and extracted the largest connected component (main component) that shows the group of highly active authors focused on grasslands at the national level (subnetworks of authors that are maximally connected between each other). We also calculated the *degree centrality* to find out which authors within the network are the most collaborative. We then compared the results with the *betweenness centrality* to highlight the most important actors within the network: respectively, authors with high degree and betweenness centralities [42, 43], metrics that represent the authors who have the most collaborative research links (and, thus, a large number of published papers) as well as authors who have a favorable position in the network (conferring on them the role of brokers in the research network).

To better understand the motivations for scientific collaboration within the field of Romanian grasslands research, three authors holding key positions in the research network were purposely sampled and asked to respond to an interview on the opportunities and constraints of research in this field. We interviewed a top Romanian author affiliated with a Romanian institution (*network leader a*, for a local perspective on grasslands research), a foreign author continuing scientific work in Romania (*network leader b*, for a perspective on opportunities for foreign scientists to develop research in Romania), and a foreign author who works abroad (*network leader c¸* for a perspective on the internationalization of grasslands research). The sampling is not probabilistic, and the results can be interpreted only as a perspective on the motivations, challenges, opportunities, and roles of domestic and foreign scientists in advancing grasslands research.

### Keywords network

Analyses based on keywords have been applied to various techniques, such as text mining, data reduction, and clustering [42, 44, 45], to identify emerging research. Keywords are also representative of the main topics addressed by research in general [46], and co-occurrence keywords network analysis is usually used to highlight the most common and important research keywords [42]. Hence, to infer the most common, popular, and bridge keywords featured in Romanian grasslands research, we used the *degree*, *eigenvector*, and *betweenness centralities* (see affiliations networks for details about these network-level metrics).

Network analyses were performed using UCINET software [47], and the networks were graphically represented using Netdraw [48] and the *chorddiag* R package [41].

For this type of study, the formal consent of the Research Ethics Committee of the University of Bucharest (https://cometc.unibuc.ro) is not required (since there was no collection or disclosure of personal information from surveys, interviews, or other personal data collection forms).

## Results

### Affiliations network

The network of organizations hosting researchers who have published work about grasslands in Romania includes 192 distinct institutions from 36 countries, out of which 14 are isolated institutions (i.e., only collaborating within themselves). Nine of the isolated institutions are foreign (e.g., Japan, Poland, Italy, Spain, Germany, Switzerland, Ukraine), but there are also 5 isolated institutions in Romania: Politehnica University of Timisoara, the University of Pitesti, the Technical University of Cluj-Napoca, the University of Bogdan Voda, and the Danube Delta National Institute for Research and Development. The best-represented institutions involved in Romanian grassland research are in Romania (53), Germany (23), the United Kingdom (13), and Hungary (13).

**Fig 3:**
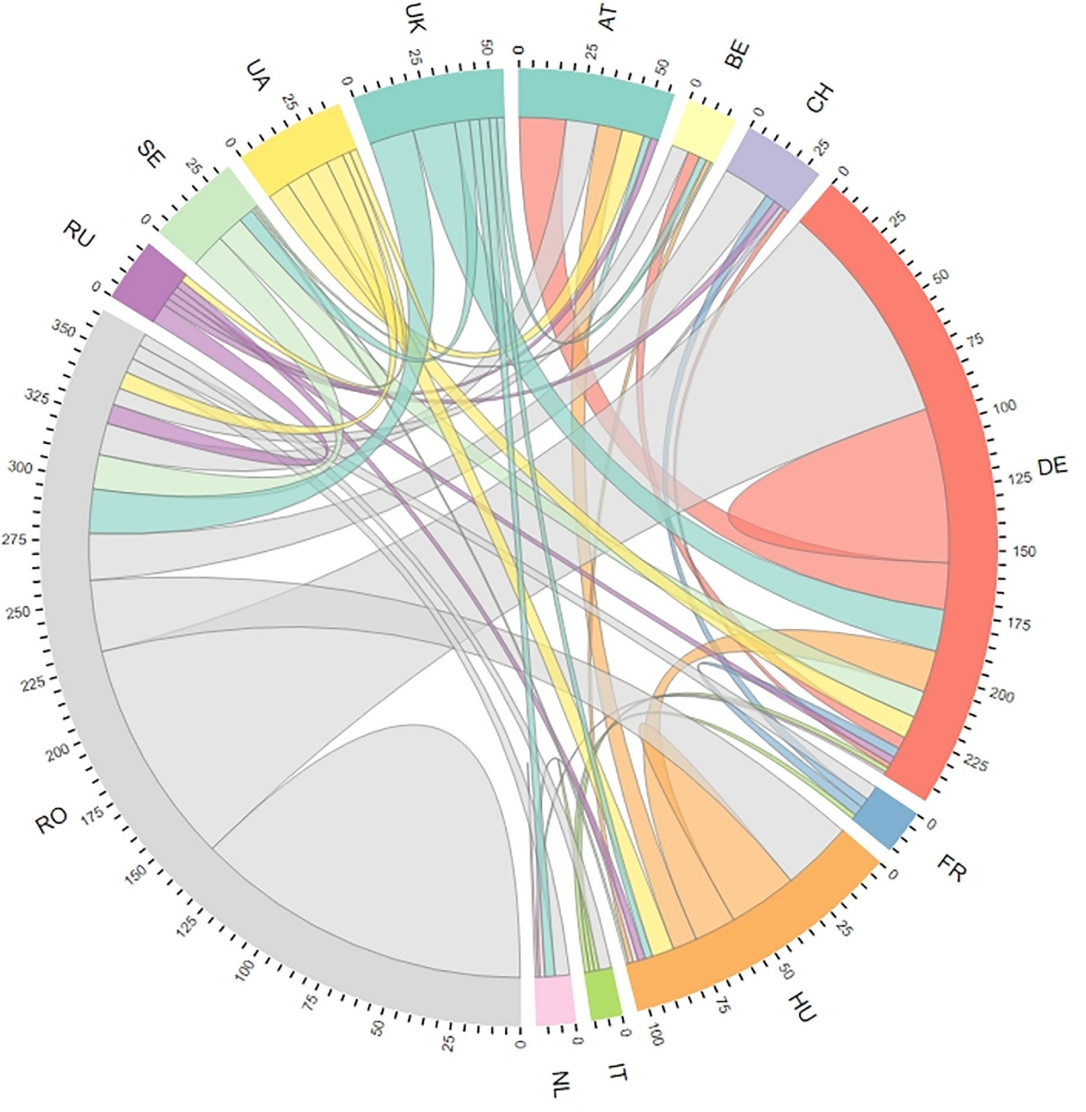
Collaborative patterns resulting from analyses of internationally visible publications related to grasslands in Romania. Links = co-occurring countries in an article; only countries with > 10 collaborations with institutions from Romania are shown.

Researchers from Romanian institutions tend to collaborate mostly with other researchers from Romania (138 institutional collaborations), followed by those from Germany (92), Hungary (29), the Czech Republic (19), and the UK (18). Researchers from the next best-represented country, Germany, collaborate mostly with institutions in Romania and Germany (Fig 3).

**Fig 4:**
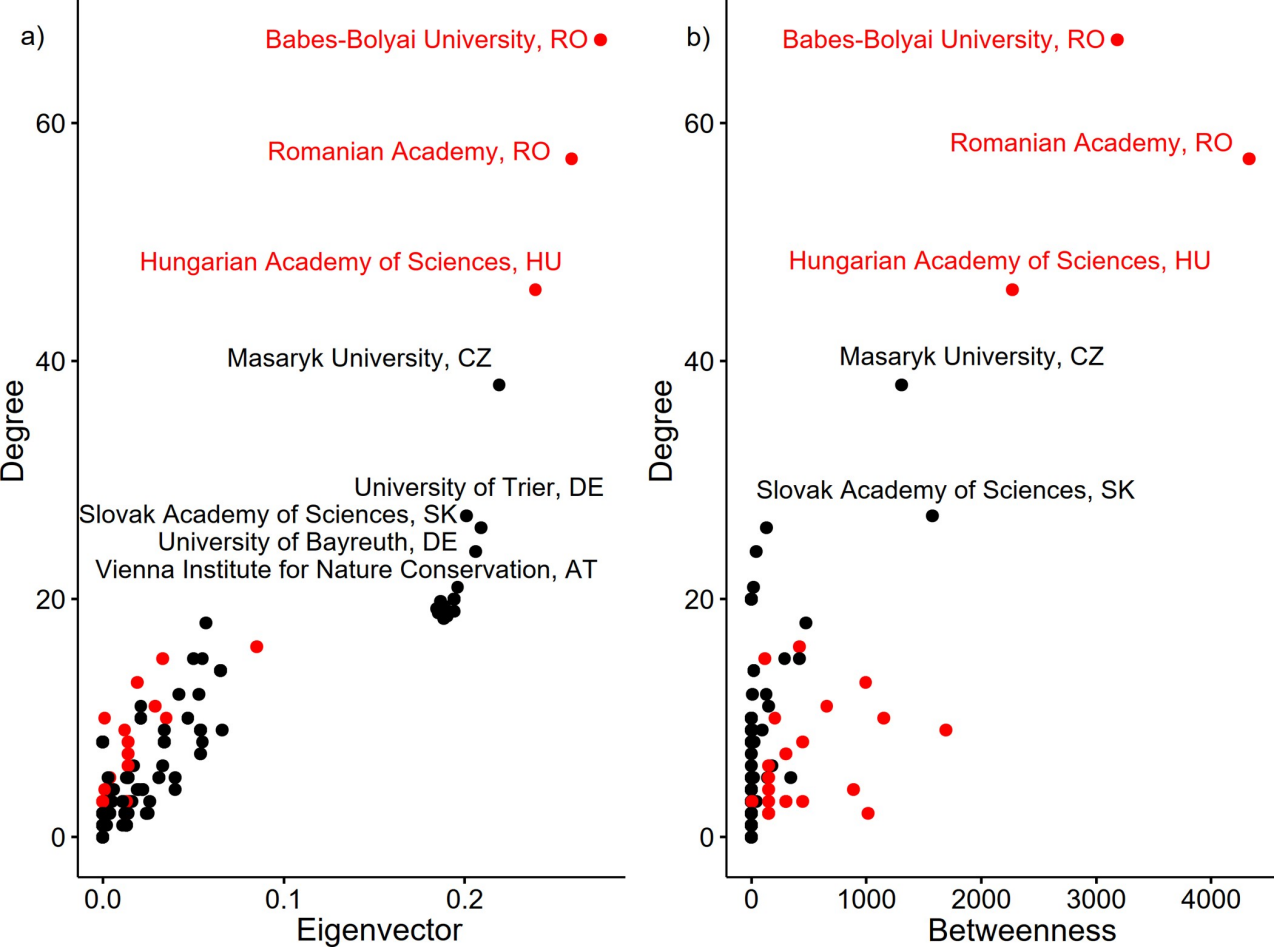
Centralities of institutions hosting researchers who have published about Romania’s grasslands (Cut-points = red).

The Bi-components analysis generated 45 blocks (S1 Table) held by 25 cut points/affiliation brokers (Fig 4) that would result in division of the structure of the network into unconnected parts if removed. Block 31 has the highest number of affiliations (101, out of which 20 are from Romania). The institutions acting as affiliation brokers are from Romania (16 institutions), Hungary (3), Slovenia (1), the Czech Republic (1), Germany (2), Sweden (1) and Italy (1).

**Fig 5:**
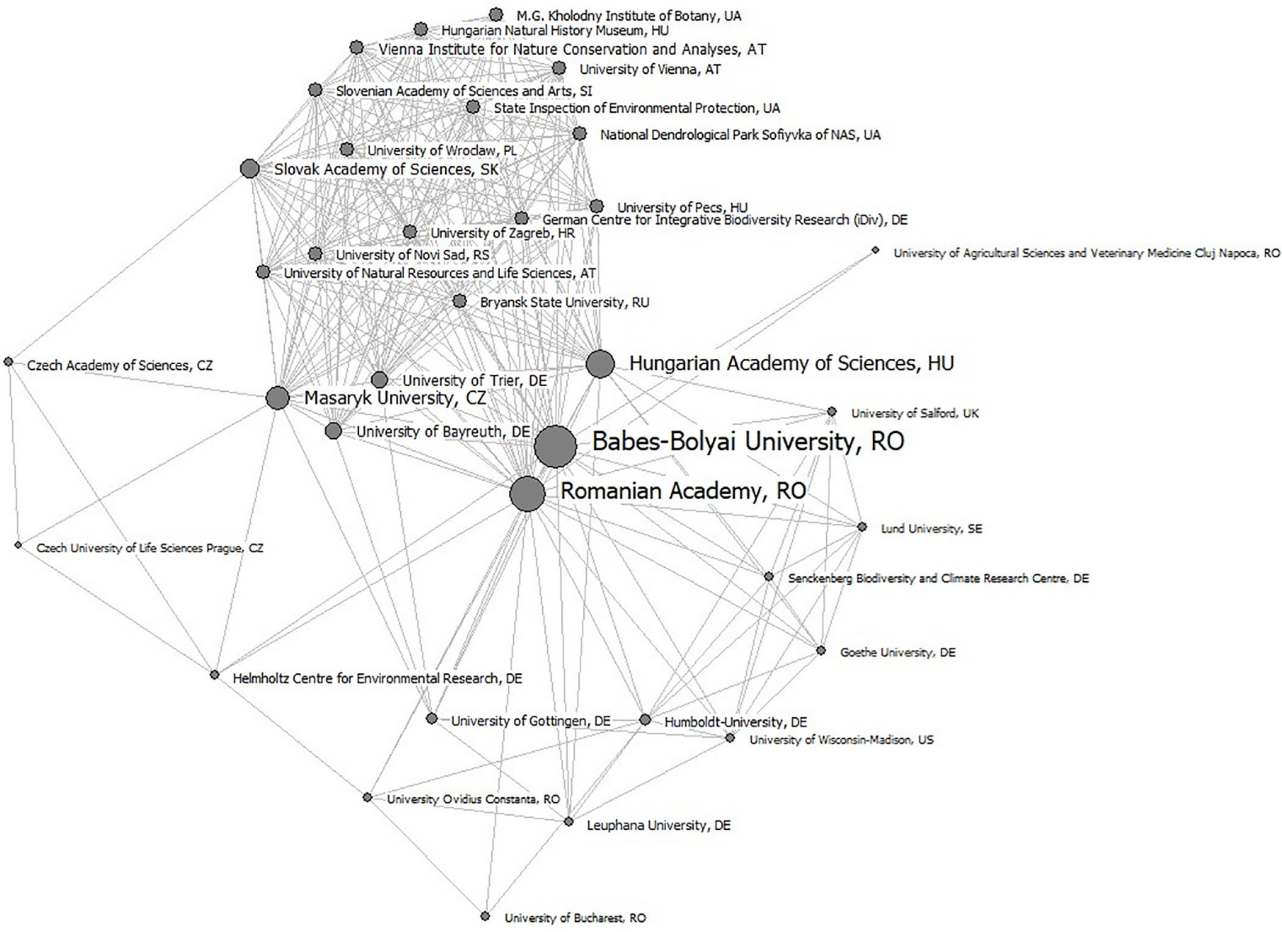
Network of the affiliations of authors publishing work about Romania’s grasslands. Size of nodes and labels are given by degree. Only affiliations with a degree centrality > 10 are shown.

Fig 4 presents the affiliations, ranked by their importance in terms of best position within the bibliometric network (betweennesses centrality), influence (eigenvector centrality), and number of network connections relevant to research of Romanian grasslands (degree centrality). The *Babes-Bolyai University* (Romania) controls the flow of information (betweenness), holds the highest number of connections (degree), and it is the most influential institution (eigenvector) within the network (S1 Table). The *Romanian Academy* (Romania) also has control over the entire network from the point of view of scientific cooperation, and it occupies the second position in terms of its number of connections and influence (Figs 4 and 5), while the third position is occupied by the *Hungarian Academy of Sciences* (Hungary).

### Network of authors

**Fig 6:**
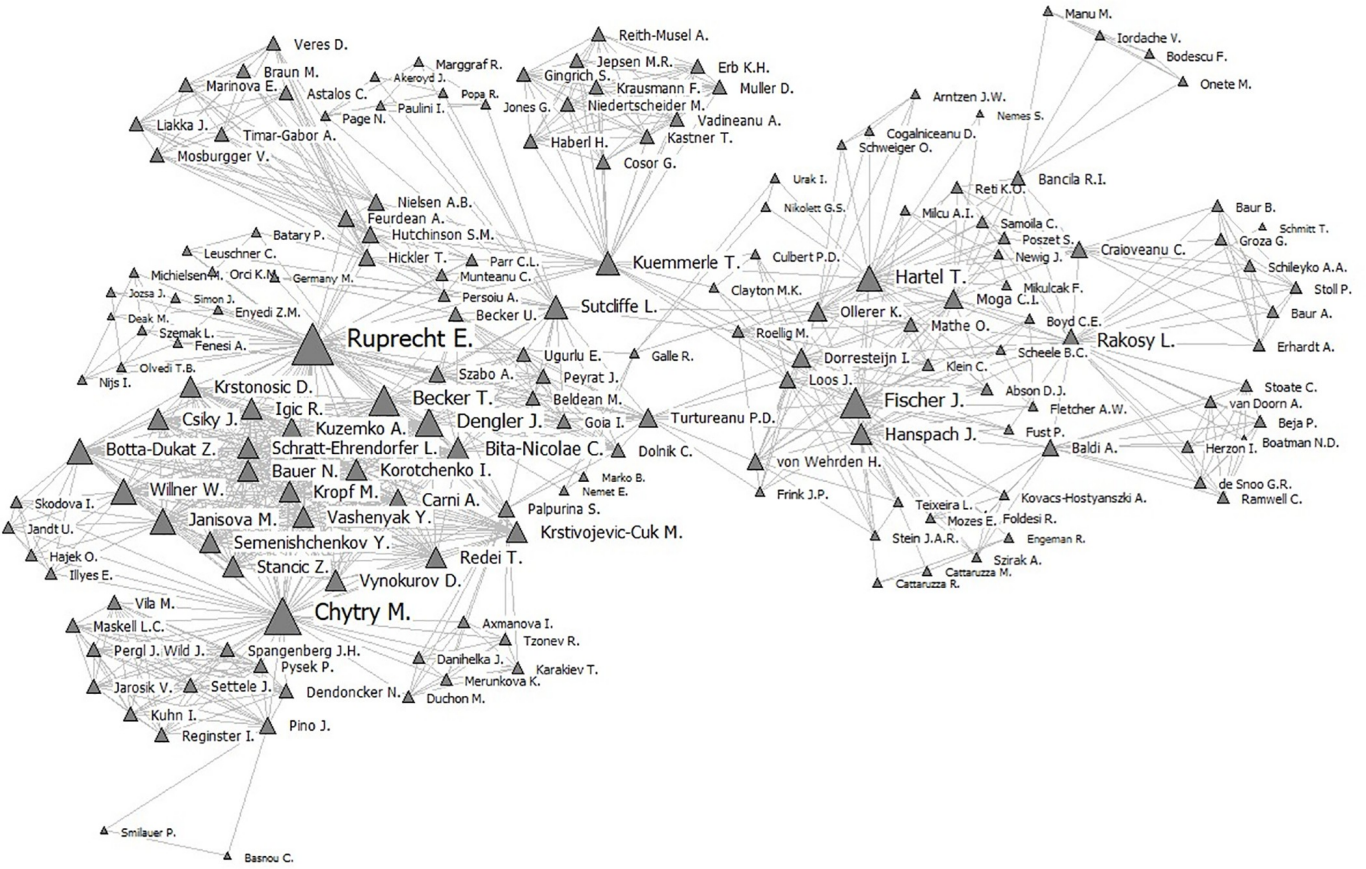
Main components of the network of authors publishing work about Romania’s grasslands (nodes size by degree).

**Fig 7:**
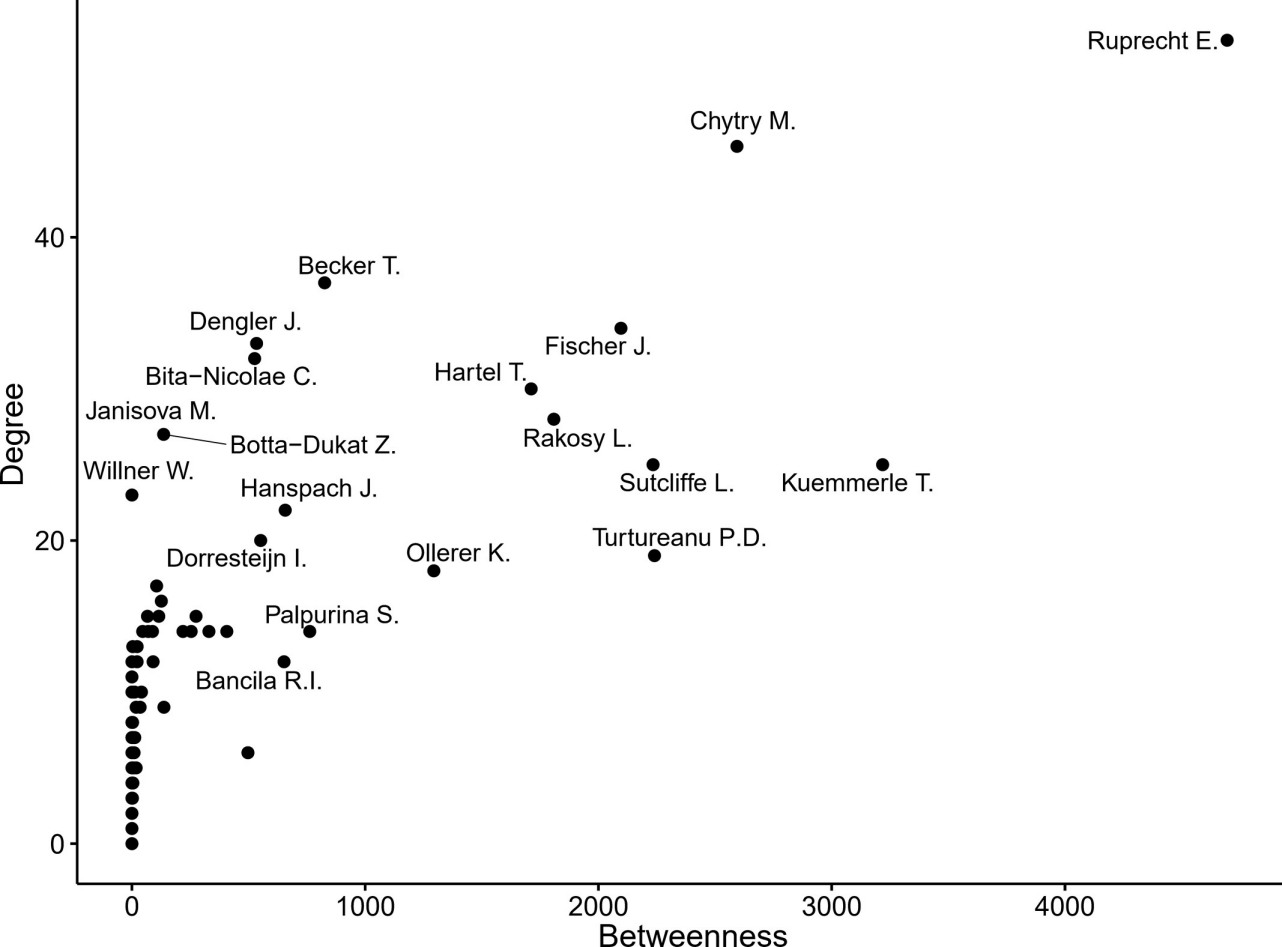
Top academic authors publishing work about Romania’s grasslands by degree and betweennesses.

The network of authors is composed of 517 authors and is characterized by a high degree of fragmentation (0.886). The main component analysis divided the fragmented network into 92 clusters (S1 Table). Cluster 6 is the densest component and includes 168 authors (Fig 6), while the rest of the clusters have a mean of 3.83 authors (stdev = 3.97). Given its importance, we further mapped Cluster 6 to examine its structure more closely (Fig 6). Most of the authors forming part of the main component are not Romanians; hence, we can see that many important authors within the Romanian grasslands research network are foreigners (Fig 7).

Based on the results, we carried out a short interview with three network leaders in order to find out how they have evolved in their professional careers, who inspired them to research the grasslands of Romania, and what constitutes the key to their success in terms of national and international partnerships in this field. The authors were anonymized as “*network leader a”* (NLa), “*network leader b*” (NLb), and “*network leader c*” (NLc).

The paragraph below summarizes the answers of the network leaders who were interviewed.

**Q1. Who/what inspired you to research grasslands in Romania (e**.**g., researchers, articles, institutions (Romanian or foreign))?**

**NLa:** *After finishing my studies at the Babeș-Bolyai University in Cluj-Napoca*, *Romania*, *I met a Romanian grassland researcher with great expertise and a passion for rare plants of steppe habitats*. *Field trips to beautiful steppe-like grasslands and the concern about their transformation or loss inspired me to study grasslands*. *Later on*, *my Ph*.*D*. *supervisor*, *especially his way of thinking about diversity*, *inspired me a lot; thus*, *I have worked with him and some of his colleagues for several years*.

**NLb:** *A friend was researching High Nature Value Farmland in Europe and told me about the species-rich grasslands in Transylvania that she had visited*. *I was curious*, *and with her assistance and the help of local partners*, *I was lucky enough to be able to do some fieldwork for a scientific study there*.

**NLc:** *It was in 1997*, *before I studied Biology*, *when I first came to Transylvania*. *I stayed for a few days with some friends in a small village not far from Sighisoara*. *It was summer*, *and one evening we climbed a hill behind the houses to watch the sunset*. *I don't remember the sunset*, *but I do remember the meadows we walked through*. *They were so amazing*. *Although I grew up on a farm myself*, *I hadn’t seen*, *nor smelled*, *nor heard such things in a meadow before*. *Every plant seemed to be blooming and every insect buzzing*. *It was such richness and beauty*. *That was the thing that made me most enthusiastic about grasslands in Transylvania*, *even if I only later learned to put scientific labels on that richness*.

**Q2**. **How did you form your co-authorship or partnership network? What were the main challenges and opportunities in this respect?**

**NLa:** *At the very beginning of my career*, *personal contacts were the most important in finding mentors*, *since there was a limited access to scientific literature*, *and even to the internet*, *in my home country in the 1990s*. *These first mentors gave me opportunities to further develop my “partnership network” by inviting me to their institutions abroad (in Hungary*, *where I had no language or cultural barriers) and to conferences*, *where I could meet other scientists*. *Later on*, *I contacted the scientists from abroad (outside of Hungary) whose papers raised my interest the most by e-mail*, *and looked for scholarships for research stays at their host institutions*. *Nowadays*, *there are scientists from European institutions contacting me and asking me for collaboration within the framework of scientific projects*.

**NLb:** *The Romanian conservation NGO Foundation ADEPT generously hosted my work in Romania*, *and through them*, *I met many*, *many interesting people involved in all aspects of conservation (academic and non-academic)*. *Perhaps the most difficult thing was to stay in touch with all of them*!

**NLc:** *My network developed through a socio-ecological research project that included research on vascular plant diversity in the farming landscape of Southern Transylvania (Sighisoara)*. *That means through multiple pathways*, *including cooperation with local NGOs*, *scientists*, *field assistants*, *and friends*. *These opportunities clearly included learning from the knowledge and experiences of others and combining our skills to get fieldwork and analysis done*. *The language barrier and the fact that we didn't do a classic vegetation study but applied a random sampling design for selecting survey sites (which didn’t match with the more traditional vegetation research) were challenges*.

**Q3**. **How would you describe the collaboration between Romanian grassland researchers?**

**NLa:** *There are few collaborations*, *and the majority are based on personal acquaintances*. *Confidence and convenience (people working at the same institution) have important roles in forming research teams around a certain project*.

**NLb:** *I only have experience of my own collaboration with Romanian grassland researchers*, *which was pleasant and productive*.

**NLc:** *To be honest*, *I don't feel I can say much about this*. *I don't have much insight into their collaboration*. *Also*, *I don't consider myself an expert on grasslands; and therefore*, *I am not so deep into this network*.

**Q4**. **How would you describe the role of foreign scientists in Romanian grasslands research?**

**NLa:** *Foreign scientists find Romanian grasslands remarkable*, *and they come to work in Romania with very specific research questions*. *They often look for a local expert in Romanian grasslands to involve in their project*, *and*, *by this means*, *collaborations arise*. *I consider that foreign scientists bring in many interesting research questions*, *achieve nice results*, *and invigorate Romanian grasslands research*.

**NLb:** *My impression is that scientists from Northern and Western Europe are very aware of the grassland diversity that has been lost in the more intensified regions of Europe*. *I think that in any ecosystem there is always value in combining observations from local researchers who intimately know the area and can interpret its subtleties and “outsiders” who can maybe bring a fresh perspective and spot parallels with other systems*.

**NLc:** *From my limited perspective*, *they seem to have a strong influence*, *or at least they seem to stand out for me*. *I could name a few foreign grasslands researchers but would struggle to name the same number of Romanians*. *However*, *this is probably biased because I am a foreigner myself*. *I might pay more attention to fellow foreigners who are active in this field*.

### Keywords network

**Fig 8:**
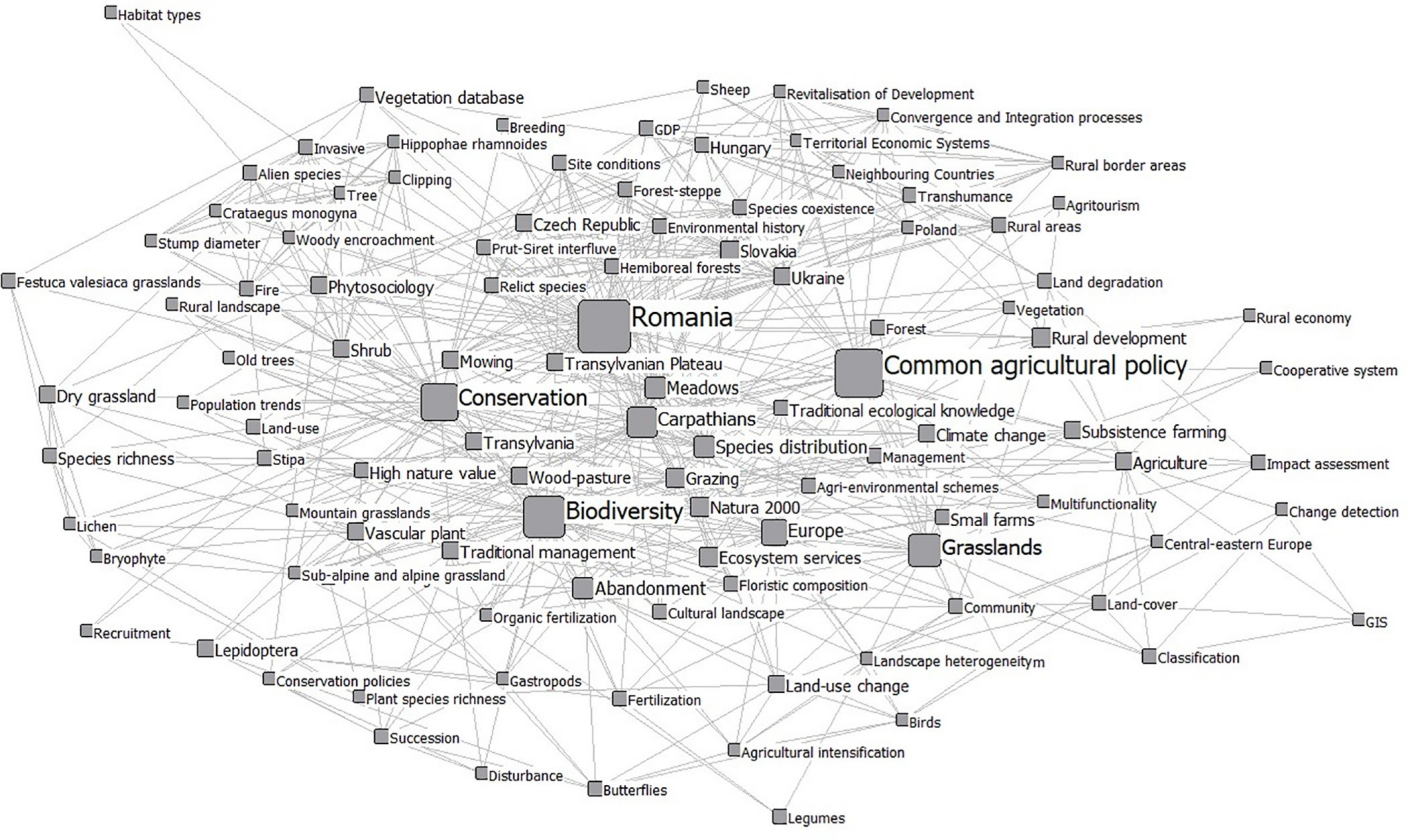
Top keywords used in publications about Romania’s grasslands by degree. Size of labels and nodes by degree; for a better visual representation, we include only keywords with a degree > 10.

**Fig 9:**
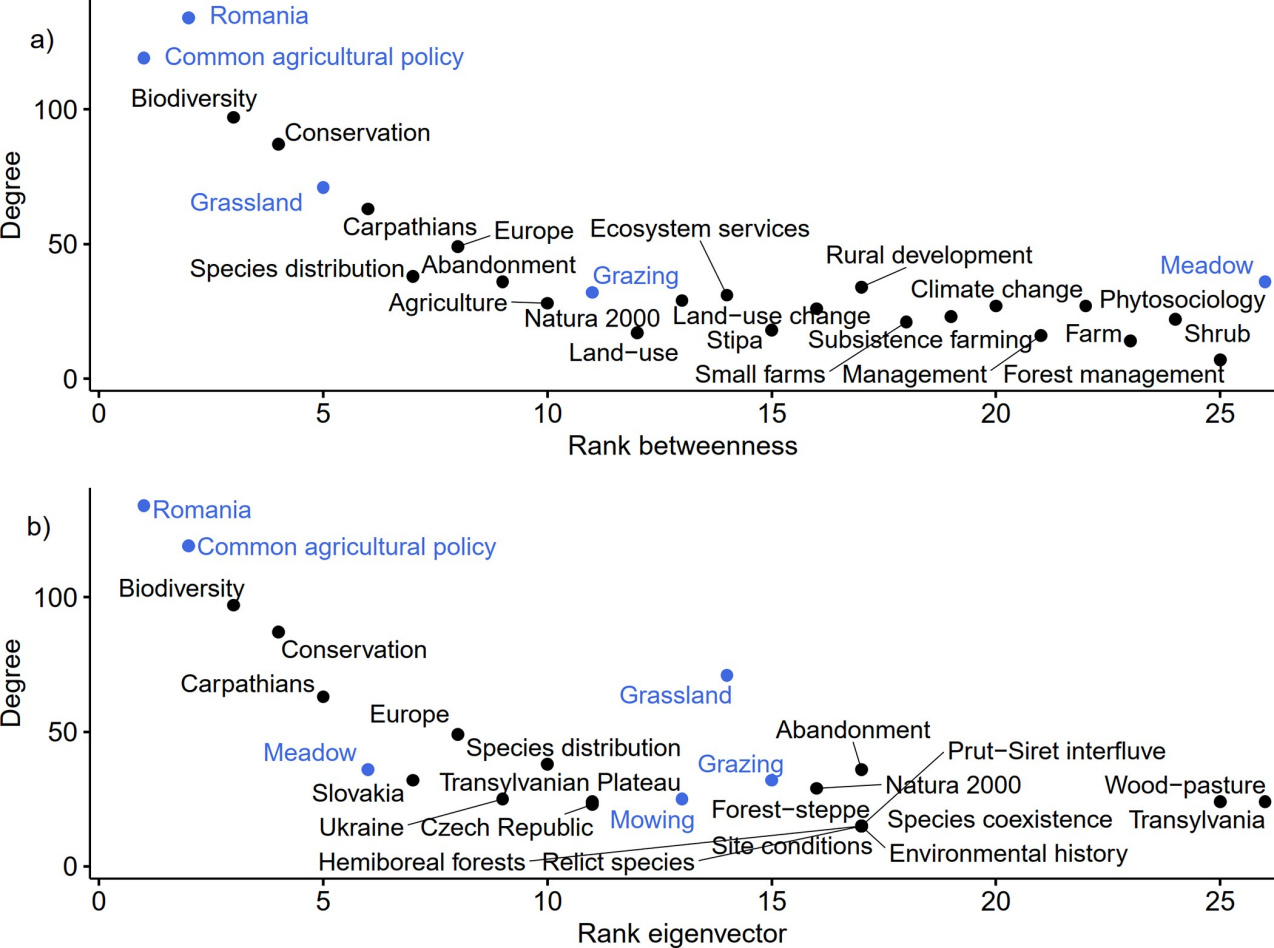
Top 25 keywords by degree and eigenvector used in publications about Romania’s grasslands. Keywords in blue were used for data collection.

To find the most-researched topics focused on grasslands, we mapped the keywords network after excluding infrequently used keywords, i.e., those with a degree of less than 10 (Fig 8). After excluding the keywords that were used to search for the articles, the most influential and important keywords within the network were *Biodiversity* and *Conservation* (Figs 8 and 9). In addition, our results showed that terms such as *Farm management*, *Pastoral value*, *Landscape pattern*, and *Ancient trees* are among many other keywords in the bottom positions, both from the perspective of their use and their importance within the network (S1 Table).

## Discussion

We provide a comprehensive analysis of the grasslands research network in an Eastern European country, Romania. Grasslands have great socio-cultural, economic, and natural value and can be approached from several disciplinary perspectives [4]. Thus, research on grasslands is an important contribution to the general knowledge because these ecosystems can be understood from multiple perspectives, including those of agronomy, nature conservation, ecology, sociology, statistics, sociology, anthropology, economics, political science, and geography. We found that both national and international researchers and institutions have contributed to the scientific knowledge of Romanian grasslands, with a high level of influence from foreign researchers. Furthermore, we identified the most commonly addressed and influential research topics related to Romanian grasslands. Nevertheless, future work must be done in order to obtain a clear image of the scientific literature focused on grasslands management at the national level, including an in-depth review.

### Who is researching Romanian grasslands?

Our results show good international participation among the institutions and researchers involved in Romanian grasslands research (192 institutions, 517 coauthors, and 197 articles). By analyzing the co-authorship centrality metrics, we discovered that the contribution of foreign institutions and researchers to grasslands research is high and that there are also key institutions in Romania that act as brokers. We are aware that a single author can name several host institutions in a research paper; however, the institution listed first is usually the home institution [43], which reduces the potential for bias. This redundancy (i.e., one author feeling a responsibility to indicate multiple institutional addresses) also may represent opportunities for innovative knowledge generation that can contribute to the cumulative knowledge base [30, 37].

Top academic authors publishing work on Romania’s grasslands in terms of number of connections (degree) and position in the network (betweenness) (Fig 7) indicated that a researcher can make an outstanding contribution to the knowledge of Romanian grasslands not only as a “grasslands specialist” but also as an ecological modeler, trans-disciplinary researcher, or economist (Fig 7) (see the interviews for the profiles of three top researchers highlighted by our analysis) within the context of a larger inter-disciplinary research group. While internationally visible scientific research production sharply increased to a maximum of 42 papers in 2017, relatively few papers were published in top-tier journals, as they have been in other Eastern European countries [49]. The lack of improvement in publishing performance can be explained by limited funding [24, 25] as well as by established traditions in choosing target journals [50]. Inter-disciplinary knowledge and international expertise are important drivers of knowledge generation [51] in grasslands management, and researchers in Romania could capitalize on this more in the future. International collaborations are increasingly becoming possible and have been encouraged (e.g., within Horizon 2020 and Biodiversa-EraNet project partnerships) [52]. These collaborations can encourage the flow of knowledge, the development of new co-authorship networks [31], and they can create a buffer for the unstable funding situation that characterizes Romanian research [24]. Through the opinions that top researchers offered in the interviews, we can form hypotheses about several ways that a local researcher might increase the attractiveness of Romanian grasslands as a subject for international research projects, including promoting a keen interest in a holistic understanding of cultural landscapes (of which grasslands are a part) and active, long-term engagement with local stakeholders and partners as well as increased scientific productivity [53]. Irrespective of the origin of the interviewee, the interviews of the three top researchers from the network indicate that cooperation in the field of Romanian grasslands research is fostered by personal recommendations and common knowledge and that the role of foreign authors and institutions is of definitive importance when forging research initiatives and partnerships. However, the interviews reflect only personal viewpoints, and they cannot be generalized to the entire community of researchers because of the potential bias of the opportunity sampling technique.

### What were the research topics addressing Romanian grasslands?

We identified 577 keywords in internationally visible research addressing Romanian grasslands. A few keywords had high importance in the overall keyword network; these could be interpreted as the main topics driving grasslands research in Romania. These keywords (Figs 8 and 9) are mostly related to the high natural value of grasslands, ecosystem services, and the land-use practices (including abandonment) related to grasslands. The results can be explained in multiple ways.

First, the accession of Romania to the European Union (2007) generated momentum, leading to the delineation of Natura 2000 sites and the development of management plans for them, which remains an ongoing process [54]. Second, conservation biology was established as a research discipline in the Romanian academic context (in addition to classical ecology research, especially during the 2000s, which also resulted in research projects that targeted rare species and habitats as well as the negative impact of management (especially overgrazing) on them). Third, several Non-Governmental Organizations also promoted social awareness of the decline of biodiversity (especially within protected areas, but also beyond them) [55], which drove research targeting the biodiversity and conservation of grasslands. Within this context, mountain hay meadows have outstanding importance (this is why the “Carpathians” were highlighted as important: Figs 8 and 9) because they are highly biodiverse and they are being threatened by overgrazing and abandonment [12, 15, 19]. A relatively recent overview of the research targeting Natura 2000 sites in the European Union showed that the research supporting the Natura 2000 network has been dominated by ecological research, while research on the policies and social elements of the network has lagged [44].

Our analysis of Romanian grasslands research suggests a similar pattern. The keywords centrality metrics identified several research topics that we would expect to be better-represented (i.e., more influential) within the network because of their crucial importance in the management of the grasslands. Some less-important keywords from the network analysis perspective are *Economy of grasslands*, *Traditional ecological knowledge related to grasslands*, *Stakeholders*, and *Non-herbaceous elements across the grasslands*. There have also been several recent research projects in different regions of Romania that aim to contribute to a socially and ecologically sustainable farming system from the perspective of holistic understanding (i.e., “Sustainable Landscapes in Central Romania”) [26].

Romania boasts some of the most representative wood-pasture systems in Central and Eastern Europe [56]. Despite their common occurrence in Romania and their relative scarcity in Western Europe [7], we found a surprisingly low number of papers addressing these systems. Since wood-pastures in Romania have suffered from a lack of tree regeneration [56] and the erosion of value related to scattered trees [57], it is imperative to encourage holistic research of these systems. Based on our collective, long-term experience as researchers within the Romanian academic system, we believe that Romanian academia still has a long way to go to encourage holistic, trans-disciplinary research to address sustainability problems related to farming landscapes in general and pastures in particular. One major barrier to adopting an integrative approach is the strong tradition of disciplinary research that still dominates research and teaching at Romanian universities. This is also reflected in the dominant research themes identified in Figs 8 and 9.

## Conclusions

Although grasslands are complex socio-ecological systems that can be studied in the context of several scientific fields and approached from an interdisciplinary perspective, internationally visible research networks related to Romania’s grasslands are still scarce (i.e., there are relatively few papers on the topic in top-tier journals and few visible researchers with institutional affiliations in Romania). The co-authorship network structure reveals several institutional leaders who can further promote research in this area. The most prestigious Romanian institutions are closely followed by foreign collaborators (Hungary and Germany). Based on their academic profiles, top researchers in the field come from diverse scientific backgrounds (plant ecology, conservation biology, population ecology, etc.), a trend that improves scientific understanding by increasing the interdisciplinary nature and relevance of research. The subjects of research are mainly related to the biological and ecological characteristics of grasslands. From the perspective of the co-occurrence of keywords, *Management of grasslands* is a notable absence from internationally visible research, especially in the context of EU Common Agricultural Policies. To improve scientific understanding and better inform EU and local policies on grasslands management, Romanian researchers should better capitalize on international collaborations and local academic leaders.

## Supporting information

S1 Table Results of network analyses of internationally visible research related to grasslands in Romania. (XLSX)
